# A high-throughput transient gene expression system for switchgrass (*Panicum virgatum *L.) seedlings

**DOI:** 10.1186/1754-6834-3-9

**Published:** 2010-05-07

**Authors:** Xinlu Chen, Raymie Equi, Holly Baxter, Kyle Berk, Jin Han, Sujata Agarwal, Janice Zale

**Affiliations:** 1 University of Tennessee, Department of Plant Sciences, 2431 Joe Johnson Drive, Knoxville, TN, 37996, USA

## Abstract

**Background:**

Grasses are relatively recalcitrant to genetic transformation in comparison to certain dicotyledons, yet they constitute some of the most important biofuel crops. Genetic transformation of switchgrass (*Panicum virgatum *L.) has previously been reported after cocultivation of explants with *Agrobacterium *and biolistics of embryogenic calli. Experiments to increase transient gene expression *in planta *may lead to stable transformation methods with increased efficiency.

**Results:**

A high-throughput *Agrobacterium*-mediated transient gene expression system has been developed for *in planta *inoculation of germinating switchgrass seedlings. Four different *Agrobacterium *strains were compared for their ability to infect switchgrass seedlings, and strain AGL1 was found to be the most infective. Wounding pretreatments such as sonication, mixing by vortex with carborundum, separation by centrifugation, vacuum infiltration, and high temperature shock significantly increased transient expression of a reporter gene (GUSPlus, a variation of the β-glucuronidase (GUS) gene). The addition of L-cysteine and dithiothreitol in the presence of acetosyringone significantly increased GUS expression compared with control treatments, whereas the addition of 0.1% surfactants such as Silwet L77 or Li700 decreased GUS expression. 4-Methylumbelliferyl beta-D-galactopyranoside (MUG) assays showed a peak of β-glucuronidase (GUS) enzyme activity 3 days after cocultivation with *Agrobacterium *harboring pCambia1305.2, whereas MUG assays showed a peak of enzyme activity 5 days after cocultivation with *Agrobacterium *harboring pCambia1305.1.

**Conclusion:**

*Agrobacterium *strains C58, GV3101 and EHA105 are less able to deliver transfer DNA to switchgrass seedlings (cultivar Alamo) compared with strain AGL1. Transient expression was increased by double or triple wounding treatments such as mixing by vortex with carborundum, sonication, separation by centrifugation, and heat shock. The addition of thiol compounds such as L-cysteine and dithiothreitol in combination with acetosyringone during cocultivation also increased transient expression. The combination of multiple wounding treatments along with the addition of thiol compounds during cocultivation increased transient expression levels from 6% to 54%. There were differences in temporal GUS expression induced by pCambia1305.1 and pCambia1305.2.

## Background

Perennial lowland switchgrass (*Panicum virgatum *L.) was chosen by Oak Ridge National Lab as a herbaceous biofuel crop of choice in 1991 because of its relatively high biomass yields in a number of replicated trials across seven states in the USA [1]. Switchgrass can be propagated by seed, survives drought better than *Miscanthus *[2], and has the ability to grow on marginal land with low fertility requirements, increasing its attractiveness in southeast USA.

Most of the economically important monocots have been relatively recalcitrant to genetic transformation compared with some dicots [3]. Transient gene expression [4,5] and stable genetic transformation of embryogenic calli in switchgrass have been reported [6-8]. *In planta *transformation of germinating cereal seedlings has been demonstrated after needle inoculation [9,10], and after shoot excision with no callus phase [11].

Several wounding treatments and additives have been shown to increase *Agrobacterium*-mediated transient gene expression and stable genetic transformation. Thiol compounds [12,13], sonication-assisted *Agrobacterium *transformation (SAAT) [14], a combination of SAAT and vacuum infiltration [15], heat and separation by centrifugation [16], surfactants [17], and mixing by vortex with carborundum [6] have been applied to a variety of explants of different species in an effort to increase transient gene expression and hence stable transformation of plants.

Transient gene expression systems are ideal for testing and comparing genetic constructs; however, increases in transient gene expression does not have a definite correlation with an increase in the production of stable transformants. Alpeter *et al*. concluded in 1996 that transient gene expression was not correlated with stable transformation in wheat [18]. However, in other studies, increased numbers of transgenic wheat and corn were regenerated from dissected explants after optimization of transient expression from reporter genes [17,19]. Transformation efficiencies of soybean and Ohio buckeye were also increased after optimization of transient expression [14].

In this paper, we describe optimization experiments and wounding treatments that significantly increased transient expression of a commercial reporter gene (GUSPlus, a variation of the β-glucuronidase (GUS) gene) in germinating switchgrass seedlings. The optimization experiments determined the most favorable *Agrobacterium *strain and acetosyringone concentrations. Wounding treatments such as sonication, mixing by vortex with carborundum, vacuum infiltration, needle wounding, separation by centrifugation, heat treatments, and additives such as L-cysteine, dithiothreitol (DTT), acetosyringone and surfactants were systematically tested in an effort to determine which treatment or combination of treatments increased transient GUS expression and the likelihood of producing stable transformants in switchgrass.

## Results and discussion

### Comparisons of different Agrobacterium strains and acetosyringone concentrations

Four different *Agrobacterium *strains (AGL1, C58, GV3101 and EHA105) were tested for their ability to deliver transfer (T)-DNA to dehusked, 3-day-old switchgrass seedlings at various acetosyringone concentrations (0, 50, 100 and 200 μM). All seedlings were treated with one of four *Agrobacterium *strains, sonicated for 1 minute, incubated for 30 minutes and then cocultivated with or without different concentrations of acetosyringone for 3 days. Seedlings were inoculated with each strain, which harbored the vector pCambia1305.2, and after cocultivation for 3 days, the number of GUS-positive seedlings was assessed. pCambia1305.2 carries the CaMV35S promoter:GRP signal peptide: catalase intron: *GUSPlus*: nos terminator [20]. The *GUSPlus *gene was originally isolated from a *Staphylococcus *species and is more stable at higher temperatures and in fixatives than the *β*-*glucuronidase *gene cloned from *Escherichia coli *[20]. The microbial glycine-rich signal peptide (GRP) from the *lac*Z alpha fragment permits secretion of GUS from the cytoplast into the apoplast [20].

The optimal *Agrobacterium *strain and acetosyringone concentrations were determined for the infection of switchgrass seedlings. The higher concentrations of acetosyringone (100 and 200 μM) induced more than double the number of GUS-positive plants, thus 100 μM was used in all experiments (Table 1). The *Agrobacterium *strain AGL1 produced significantly more GUS positive plants compared with inoculation with other *Agrobacterium s*trains (Table 1), therefore this strain was used in all subsequent experiments to optimize other parameters. Strains GV3101 and C58 were the least able to deliver T-DNA to the switchgrass seedlings.

**Table 1:** Comparison of GUSPlus expression in switchgrass seedlings induced by four *Agrobacterium *strains, at different acetosyringone concentrations.

*Agrobacterium *strains	Acetosyringone, μM				GUS-positive seedlings		
	
	0	50	100	200	Mean ± SD*	Total, n†	%
AGL1	2	6	14	14	9.0 ± 3.0^a^	36	6.0

EHA105	2	4	7	7	5.0 ± 1.2^b^	22	3.7

GV3101	2	2	2	1	1.5 ± 0.3^c^	7	1.2

C58	1	1	0	0	0.5 ± 0.3^c^	2	0.1

AS = acetosyringone; GUS = β-glucuronidase.

*Means followed by the same letter (a, b, c) were not significantly different at the 5% level using The Tukey multiple comparison test.

† Three replicates of 50 seedlings tested with each strain at each acetosyringone concentration.

### The effects of various treatments and additives on GUSPlus expression

**Figure 1:**
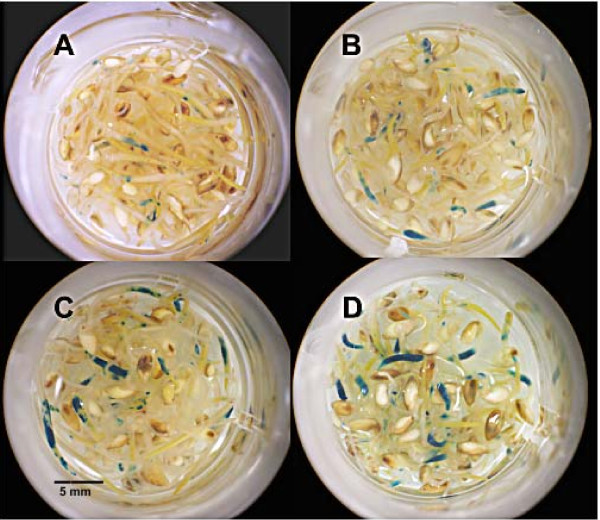
**Microtitre plate with switchgrass seedlings assayed for GUSPlus activity after treatment with or without thiol compounds**. Coculture with **(a) ***Agrobacterium *in water and acetosyringone; **(b) **with *Agrobacterium *in water, acetosyringone and L-cysteine; **(c) **with *Agrobacterium *in water, acetosyringone and dithiothreitol (DTT); and **(d) **with *Agrobacterium *in water, acetosyringone, L-cysteine and DTT.

To investigate the role that thiol compounds play in promoting gene expression, switchgrass seedlings were inoculated with *Agrobacterium *strain AGL1, sonicated for 1 minute and incubated for 30 minutes. They were then placed on filter paper with H2O and 100 μM acetosyringone, or a combination of H2O, DTT and L-cysteine with acetosyringone for 3 days of cocultivation. There were significantly more GUS foci when the *Agrobacterium*-inoculated seedlings were grown in the combination of water, acetosyringone, L-cysteine and DTT (Table 2, Figure 1). Transient GUS expression has also been increased with thiol compounds in other species such as soybean [20] and maize [19].

**Table 2:** A comparison of thiol compounds and their effect on transient gene expression of GUSPlus.

Treatment*	GUS-positive seedlings		
	
	Total, n*	Mean ± SE†	%
H_2_O, AS	21	7.0 ± 0.6^b^	14.0

H_2_O, cysteine, AS	26	8.7 ± 2.3^b^	17.3

H2O, DTT, AS	29	9.7 ± 2.2^b^	19.3

H_2_O, cysteine, DTT, AS	47	15.7 ± 1.9^a^	31.3

AS = acetosyringone; DTT = dithiothreitol.

All of the treatments included 1 minute of sonication.

*Three replicates of 50 seedlings per replicate were tested in each treatment.

†Means followed by the same letter (a, b, c) were not significantly different at the 5% level as determined by The Tukey multiple comparison test.

**Figure 2:**
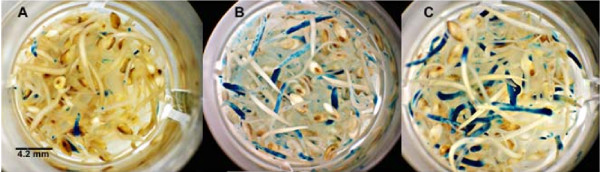
**Microtitre plate of seedlings inoculated with *Agrobacterium*, treated by sonication, needle wounding or mixing by vortex with carborundum and stained for β-glucuronidase (GUS)Plus activity**. **(a) **Control (sonicated) switchgrass seedlings; **(b) **sonicated seedlings that were needle inoculated; **(c) **sonicated seedlings that were mixed by vortex with carborundum. All seedlings were assayed for GUSPlus activity.

An experiment was designed to compare needle wounding and sonication versus mixing by vortex with carborundum and sonication to determine which wounding treatment was superior. Seedlings 3 days old were either punctured with an *Agrobacterium*-coated needle or mixed by vortex for 2 minutes with the *Agrobacterium *resuspension solution containing carborundum. All of the seedlings were then sonicated for 1 minute in he *Agrobacterium *resuspension solution. There were significant differences in GUS expression between the needle wounding, mixing by vortex with carborundum, and the control (sonication alone) groups (Table 3). Of the seedlings mixed by vortex with carborundum, 42% expressed GUS compared with 32% of seedlings wounded with a needle and only 18.7% of the control seedlings (Table 3, Figure 2). Carborundum is an abrasive silicon carbide material that induces wounding of plant tissue, and this treatment had previously been used in *Agrobacterium*-mediated genetic transformation of switchgrass [6]. Scanning electron microscopy has shown that sonication creates micro-wounds (1 μm to 1 mm in size) in embryonic suspension tissues of soybean, and it is through these pores that *Agrobacterium *enters and adheres to the cell. There was little *Agrobacterium *adherence to cells that did not receive the sonication treatment [14].

**Table 3:** A comparison of mixing by vortex with carborundum, needle wounding and sonication on GUSPlus expression.

Treatment*	GUS-positive seedlings		
	
	Total, n*	Mean ± SE†	%
Control (sonication)	28	9.3 ± 1.2^c^	18.7

VCS	63	21.0 ± 2.1^a^	42.0

NWS	48	16.0 ± 2.3^b^	32.0

NWS = needle wounding and sonication; VCS = vortex, carborundum and sonication

*Three replicates of 50 seedlings per replicate were tested in each treatment.

† Means followed by the same letter (a, b, c) were not significantly different at the 5% level as determined by The Tukey multiple comparison test.

Vacuum infiltration and separation by centrifugation treatments were applied to sonicated switchgrass seedlings in an attempt to increase wounding and *Agrobacterium *infection and ultimately transient GUS expression. Switchgrass seedlings were inoculated with *Agrobacterium*, sonicated for 1 minute, incubated for 30 minutes, vacuum infiltrated for 0, 1, 2, 4, 8 or 16 minutes, and in some treatments, separated by separation by centrifugation. All treatments were then cocultured with *Agrobacterium *for 3 days. There were significant differences in GUS expression between the treatments in which vacuum infiltration and/or separation by centrifugation pretreatments were applied, with separation by centrifugation having the greater effect (Table 4).

**Table 4:** A comparison of vacuum infiltration and separation by centrifugation on GUSPlus expression in sonicated switchgrass seedlings.

Treatment*	GUS-positive seedlings		
	
	Total, n*	Mean ± SE†	%
Control-1 (no vacuum; no separation by centrifugation)	17	4.2 ± 0.6^b^	8.5

Control-2 (no vacuum; separation by centrifugation)	43	10.8 ± 2.6^a^	21.5

Vacuum (1 minute); separation by centrifugation	43	10.8 ± 1.6^a^	21.5

Vacuum (2 minutes); separation by centrifugation	44	11.0 ± 2.1^a^	22.0

Vacuum (4 minutes); separation by centrifugation	49	12.2 ± 3.2^a^	24.5

Vacuum (8 minutes); separation by centrifugation	50	12.5 ± 0.5^a^	25.0

Vacuum (16 minutes); separation by centrifugation	55	13.8 ± 1.4^a^	27.5

+ All seedlings were sonicated for 1 minute.

*Three replicates of 50 seedlings per replicate were tested in each treatment.

†Means followed by the same letter (a, b, c) were not significantly different at the 5% level as determined by The Tukey multiple comparison test.

Two surfactants (Li700 and Silwet L77) at five concentrations (0, 0.01, 0.02, 0.04 and 0.1%) were compared to determine which was optimal for inducing transient GUS expression in germinating 3-day-old switchgrass seedlings. Silwet L77 is an organosilicone surfactant used as a wetting agent in the floral dip method [21,22] and in *Agrobacterium-*mediated transformation experiments of dissected explants [23]. Li700 is a nonionic acidifying surfactant commonly used in herbicide application, and is less phytotoxic than Silwet L77 (Zale, unpublished data). Seedlings were inoculated with *Agrobacterium *and surfactant, mixed by vortex with carborundum for 2 minutes, sonicated for 1 minute, separated by separation by centrifugation, and assayed for GUS expression. There were no significant differences between Li700 and Silwet L77 in increasing GUS expression and the highest concentration of either surfactant (0.10%) inhibited GUS expression to the greatest degree (Table 5).

**Table 5:** A comparison of two different surfactants, at five concentrations, on transient gene expression of GUSPlus.

Conc., %	LI700		Silwet L77		Combined
	
	GUS- positive seedlings, n†	GUS- positive seedlings, %	GUS- positive seedlings, n†	GUS- positive seedlings, %	GUS- positive seedlings, mean ± SE
0.0	87	43.5	89	44.5	22.0 ± 0.8^a^

0.01	93	46.5	97	48.5	23.7 ± 0.5^a^

0.02	116	58.0	99	49.5	26.9 ± 1.4^a^

0.04	68	34.0	87	43.5	19.3 ± 1.6^b^

0.10	19	9.5	16	8.0	4.4 ± 0.8^c^

Conc. = concentration.

There were no significant differences between surfactants, but there were significant differences between the concentrations of surfactants.

*Three replicates of 50 seedlings per replicate were tested in each treatment.

† Means followed by the same letter (a, b, c) were not significantly different at the 5% level as determined by The Tukey multiple comparison test.

A heat shock has been shown to increase transformation efficiencies in some crops [16], therefore four different temperatures were tested in an effort to increase transient GUS expression. The seedlings were inoculated with the *Agrobacterium *resuspension solution, mixed by vortex with carborundum, and sonicated. The tubes were placed at one of five temperatures (25, 37, 40, 43 and 46 degrees Celcius) for 2 minutes, incubated at room temperature for 30 minutes, separated by separation by centrifugation, and then plated onto filter paper for 3 days of cocultivation. There were significant differences between the temperature treatments, with heat-shock treatments producing the greatest number of GUS-positive seedlings compared with the 25°C treatment (Table 6).

**Table 6:** The effect of heat shock on transient expression of GUSPlus.

Temperature, °C	GUS-positive seedlings		
	
	Total, n*	Mean ± SE†	%
25	66	22.0 ± 1.2^b^	44.0
37	81	27.0 ± 0.6^a^	54.0
40	78	26.0 ± 0.6^a^	52.0
43	79	25.3 ± 0.9^a^	52.7
47	77	25.7 ± 0.9^a^	51.3

*Three replicates of 50 seedlings per replicate were tested in each treatment.

† Means followed by the same letter (a, b, c) were not significantly different at the 5% level as determined by The Tukey multiple comparison test.

### GUS expression comparisons between pCambia 1305.1 and 1305.2

**Figure 3:**
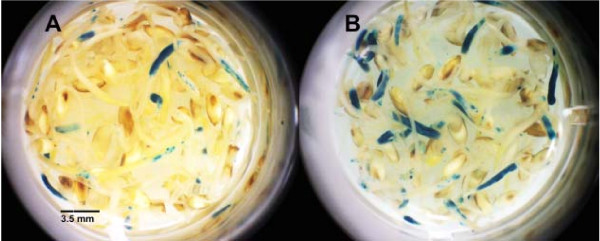
**Microtitre plate comparing switchgrass seedlings after a 3-day inoculation with *Agrobacterium *harboring two different β-glucuronidase (GUS) plasmids and assayed for GUS activity**. Switchgrass seedlings inoculated with *Agrobacterium *harboring **(a) **pCambia 1305.1 and **(b) **pCambia 1305.2.

The expression patterns induced by pCambia1305.1 and 1305.2 were compared to determine whether there was a difference in transient gene expression due to the GRP signal peptide present in the latter. After 3 days of coculture, pCambia1305.2 produced significantly more GUS activity compared with pCambia1305.1, probably because this enzyme is secreted from the cell into the apoplast, whereas the encoded GUS enzyme in pCambia1305.1 remains in the cytosol (Table 7, Figure 3). Because of the GRP signal sequence, the GUS enzyme is secreted from the cell, and this permits plant selection on tissue culture medium with nonlethal concentrations of the substrate, X-glururonide [20]. GUS expression after *Agrobacterium *cocultivation with both plasmids was localized throughout the shoot tissue rather than the roots or cotyledons, indicating that the target tissue was the shoot tissue.

**Table 7:** Comparison of GUSPlus activity between pCambia 1305.1 and 1305.2 after 3 days of cocultivation.

Treatment*	GUS-positive seedlings		
	
	Total, n*	Mean ± SE†	%
pCambia 1305.1	48	12.0 ± 1.1^a^	24

pCambia 1305.2	76	19.0 ± 2.6^b^	38

*Three replicates of 50 seedlings per replicate were tested in each treatment.

† Means followed by the same letter (a, b, c) were not significantly different at the 5% level as determined by The Tukey multiple comparison test.

**Figure 4:**
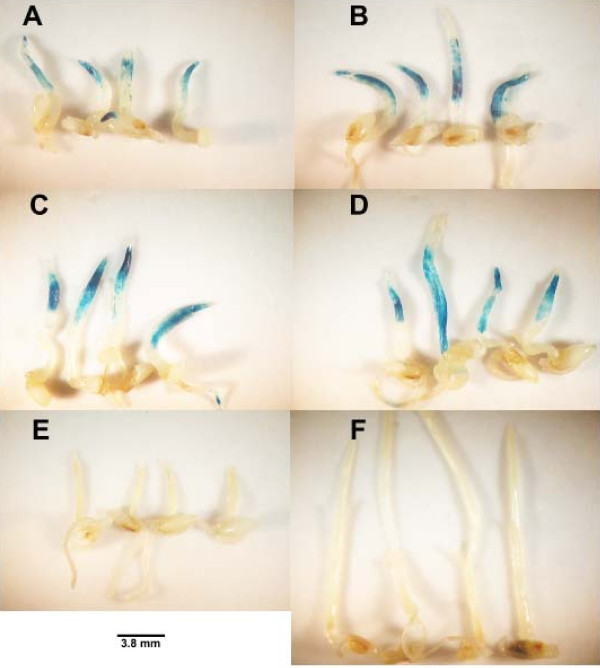
**Duration of β-glucuronidase (GUS) expression after a 3-day cocultivation with *Agrobacterium*harboring pCambia 1305.1, pCambia 1305.2 and a control**. Seedlings cocultivated with **(a, c) **pCambia 1305.1 and **(b, d) **pCambia 1305.2 at 3 and 5 days after cocultivation, respectively, and stained for GUS. **(e, f) **Control seedlings at 2 and 7 days after cocultivation without *Agrobacterium*, respectively, and stained for GUS.

To determine the onset and duration of GUS expression in seedlings inoculated with *Agrobacterium *harboring pCambia1305.1 and pCambia1305.2, seedlings were treated with all of the aforementioned significantly effective wounding treatments and additives, and histochemical GUS assays were conducted on various days (0, 2, 3, 5, 7 and 11 days) after a 3-day cocultivation. There was no GUS expression in the *Agrobacterium*-inoculated seedlings with either plasmid or the control on the first day after inoculation (data not shown). Intense GUS expression induced by pCambia1305.2 occurred at 3 days after cocultivation (Figure 3; compare Figures 4a and 4b). By contrast, intense GUS expression induced by pCambia1305.1 developed by the fifth day after cocultivation (compare Figure 4c and 4d), because this enzyme is not secreted and would accumulate within the cell over time. GUS expression induced from both plasmids occurred extensively in the shoots and less in the coleoptiles and roots. By the seventh day after cocultivation, GUS expression induced by both plasmids was reduced (data not shown).

**Figure 5:**
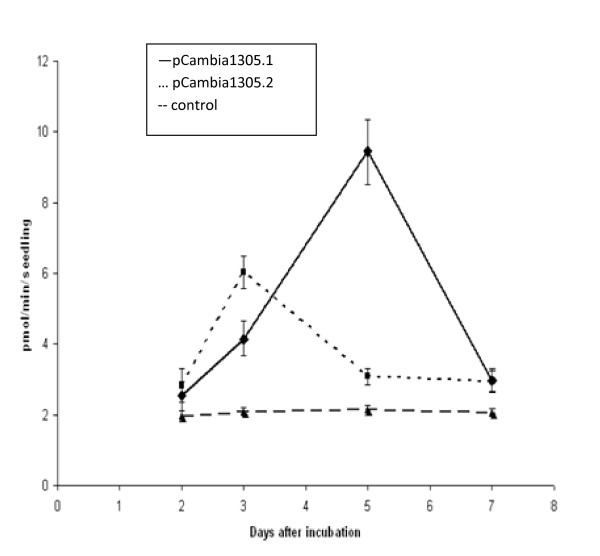
**MU fluorescence due to β-glucuronidase (GUS) activity in switchgrass seedlings days after a 3-day cocultivation with *Agrobacterium *harboring pCambia1305.1, pCambia1305.2 and a control**. The number of days after cocultivation is shown on the *x *axis, and the 4-methylumbelliferone (MU) fluorescence per seedling is shown on the *y *axis. Standard errors are shown for each measurement.

Quantitative fluorometric.4-methylumbelliferyl-beta-D-glucuronide (MUG) assays were compared between pCambia1305.1, pCambia1305.2 and control seedlings, from the second to the seventh day after cocultivation. In these assays, GUS reacts with MUG to release the fluorescent compound 4-methyl umbelliferone, and fluorescence can then be measured [24]. There were peaks of fluorescence induced by GUS on the third day after cocultivation with pCambia1305.2 and on the fifth day after cocultivation with pCambia1305.1, which agrees with the intensities of the histochemical GUS assays (Figure 4, Figure 5). Moreover, the magnitude of the fluorescence induced by the encoded enzyme in pCambia1305.1, although not statistically significant, was greater than that of pCambia1305.2.

## Conclusion

These experiments optimized the treatments, additives and *Agrobacterium *strains for transient gene expression in switchgrass. The *Agrobacterium *strain AGL1 was most able to infect 3-day-old Alamo switchgrass seedlings in the presence of 100 μM acetosyringone. Double or triple wounding treatments resulted in the highest levels of transient GUSPlus expression. The treatments that significantly increased GUS expression were sonication, needle wounding, mixing by vortex with carborundum, separation by centrifugation and heat shock. Mixing by vortex with carborundum and separation by centrifugation appeared to have the greatest effect on GUS expression in switchgrass. The addition of L-cysteine and DTT during cocultivation also significantly increased GUS expression. Transient gene expression was increased from 6% in untreated seedlings to 54% after the application of a sequential set of treatments and additives. The *GUS *genes encoded on the two pCambia plasmids, 1305.1 and 1305.2, had different temporal expression patterns in switchgrass seedlings, and histochemical and MUG assays suggest that the enzyme activity of 1305.1 accumulates in the cytosol over a longer period than that of 1305.2.

## Methods

### Plant material and preparation

Alamo seeds, purchased from the Bamert Seed Company in Muleshoe, TX, were dehusked by soaking in 60% H_2_SO_4 _for 30 minutes with shaking, and then washed six times in sterile distilled water with shaking, 5 minutes per wash. The seeds were sterilized in 250 ml of 100% commercial bleach (6% NaClO) with 0.05% of Tween-20 for 30 minutes with shaking and washed six times with sterile distilled water with shaking, 5 minutes per wash. Sterilized seeds were plated on seed germination medium composed of MS salts [25], supplemented with Gamborg B5 vitamins [26], 2% sucrose, 0.3% Gelrite (Research Products International, Mt. Prospect, IL), pH 5.8 and maintained at 24 ± 2°C in the dark.

### Agrobacterium strains, plasmid and bacterial induction

Four *Agrobacterium *strains, AGL1 [27], EHA105 [28], GV3101 [29] and C58 [30] were evaluated for their ability to infect germinating switchgrass seeds. AGL1 and EHA105 are hypervirulent strains [27,28]. All *Agrobacterium *strains harbored pCambia1305.1 or pCambia 1305.2 http://www.cambia.org/daisy/cambia/585.html[20]. These plasmids are the same except the pCambia1305.2 carries the GRP signal peptide sequence which permits secretion of GUSPlus enzyme from the cell. Both carry the catalase intron:GUS sequence to prevent expression of GUS genes in *Agrobacterium and *the hygromycin gene as a plant selectable marker. Four different concentrations of acetosyringone (0, 50, 100, 200 μM) were tested to determine which induced the virulence of *Agrobacterium *to the greatest extent as determined by GUS staining.

The *Agrobacterium *was grown in liquid YEP medium (10 g l-1 Bacto Peptone, 10 g l-1 yeast extract, 5 g/l NaCl, pH7.0) overnight and separated by separation by centrifugation the next morning at 1376× g at room temperature (22 ± 4°C) for 10 minutes. The bacterial pellet was gently resuspended in liquid resuspension solution (0.1× MS, 1× vitaminB5, 3% sucrose, 1.2 g/l 2-(N-Morpholino)ethanesulfonic acid (MES), pH 5.4) and diluted to OD_600 _= 1.0, then 1 M acetosyringone (Acrose Organics, Morris Plains, NJ, USA) dissolved in dimethyl sulfoxide, and added to a final concentration of 100 μM for a 3-hour induction period (henceforth termed the *Agrobacterium *resuspension solution).

### Treatments and Agrobacterium inoculation

To test which strain of *Agrobacterium *induced the greatest GUSPlus staining, four *Agrobacterium *strains harboring pCambia1305.2 were used to inoculate 3-day-old switchgrass seedlings. In some treatments, the junction area between the root and shoot were pierced two or three times with a sterile needle that had been dipped in *Agrobacterium *resuspension solution under a dissecting microscope (Stereomaster, Fisher Scientific, Pittsburgh, PA, USA). After wounding, the entire seedlings were placed in *Agrobacterium *resuspension solution, incubated for 30 minutes and placed onto sterile 8.4 cm filter paper in a 100 × 15 mm Petri dish (BD Biosciences, Franklin lakes, NJ, USA) wetted with either 1.6 ml of sterile H_2_O and 100 μM acetosyringone or 1.6 ml of a solution comprising H2O, DTT (154 mg/l; [12]), L-cysteine (400 mg/l; [11,12]) and acetosyringone (100 μM), incubated at room temperature in the dark for 3 days of cocultivation. Three replicates, with 50 seedlings per replicate were tested for each strain and at each acetosyringone concentration.

For the sonication treatments, 10 seedlings were placed in sterile tubes to which 500 μl of *Agrobacterium *resuspension solution was added, and the tubes were placed in a sonicator (Branson 1210; Fisher Scientific, Atlanta, GA, USA) for various durations (0, 0.5, 1, 2, 4 and 8 minutes), after incubation with *Agrobacterium *resuspension *s*olution for 30 minutes. The seedlings were spread onto sterile 8.4 cm filter paper in a 100 × 15 mm Petri dish; the paper was wetted with either 1.6 ml of sterile H2O and 100 μM acetosyringone, or sterile H2O, DTT (154 mg/l; [12]), L-cysteine (400 mg/l; [12,13]) and acetosyringone (100 μM), and were cocultured for 3 days at room temperature in the dark. There were three replicates, with 50 seedlings per replicate.

Vortex-carborundum-sonication treatments consisted of placing 50 seedlings in 50 ml tubes (Falcon; Becton Dickinson Labware, Franklin Lakes, NJ, USA) to which 5 ml of *Agrobacterium *resuspension solution and 1 ml of 0.1% carborundum solution (w/v) (Fisher Scientific, Atlanta, GA, USA; [6]) were added. The tubes were then mixed by vortex at 4000 rpm for 2 minutes. After the carborundum treatment, the seedlings were placed in 1.5 ml microcentrifuge tubes (10 seedlings per tube), to which 500 μl of the *Agrobacterium *resuspension solution was added, and the tubes were sonicated for 1 minute. The sonicated seedlings were incubated for 30 minutes at room temperature, separated by centrifugation at 2400 g, for 1 minute, spread onto filter paper and cocultivated with *Agrobacterium *as described previously. There were three replicates, with 50 seedlings per replicate.

For vacuum infiltration treatments, 3-day-old seedlings were placed in sterile 1.5 ml microcentrifuge tubes (10 seedlings per tube) to which 500 μl of *Agrobacterium *resuspension solution was added, and the tubes were placed in a sonicator (Branson 1210; Fisher Scientific) for 1 minute. The tubes were then placed in a vacuum chamber under vacuum (610 mm of Hg) for 0, 1, 2, 4, 8 and 16 minutes. This was followed by incubation with *Agrobacterium *resuspension *s*olution for 30 minutes and separation by centrifugation (1 minute at 2400 g). The 3-day cocultivation was the same as described above.

Heat-shock treatments were applied by inserting the samples (10 seedlings per microcentrifuge tube in 500 μl *Agrobacterium *resuspension solution) into a heating block at various temperatures (25, 37, 40, 43, 47 degrees Celsius) for 2 minutes, then placing the tubes into a sonicator for 1 minute (as previously described), followed by incubation for 30 minutes, and separation by centrifugation at 2400 for 1 minute. The 3-day cocultivation was as described above, using three replicates, with 50 seedlings per replicate.

For surfactant treatments, two surfactants (Li700 (Loveland Products Inc., Greeley, Colorado, USA) and Silwet L-77 (Lehle Seeds, Round Rock, TX, USA)) were compared at five concentrations (0, 0.01, 0.02, 0.04 and 0.1% v/v). The surfactants were added to the *Agrobacterium *resuspension solution in which the seedlings were placed (each tube contained 500 μl of solution per 10 seedlings). The samples were sonicated for 1 minute, incubated for 30 minutes and separated by centrifugation at 2400 for 1 minute. The 3-day cocultivation was as described above, using three replicates, with 50 seedlings per replicate.

### Histochemical and fluorometric assay of GUS expression

GUS staining was performed according to Jefferson *et al*. [24] with some modifications. The seedlings were incubated overnight at 37°C in a solution containing 50 mM sodium phosphate buffer (pH 7.0), 2 mM EDTA, 0.12% Triton, 0.4 mM ferrocyanide, 0.4 mM ferricyanide, 1.0 mM 5-Bromo-4-chloro-3-indoxyl-beta-D-glucuronide cyclohexylammonium salt (X-Gluc) (Gold Biotechnolgy, St. Louis, MO, USA) and 20% methanol anhydrate. Seedlings with GUS foci > 2 mm were counted as positive.

Fluorometric MUG assays were performed according to Jefferson *et al*. [24] with some modifications. Shoots of 50 seedlings per treatment were excised, placed in 1.5 ml microcentrifuge tubes, and homogenized with a small mortar and pestle and liquid nitrogen. After homogenization, 250 μl of extraction buffer (50 mM sodium phosphate, pH 7.0, 10 mM EDTA, 0.1% Triton X-100, 0.1% SDS, 10 mM β-mercaptoethanol) was added. The pellet was resuspended by mixing by vortex and separated by centrifugation at 10,000 at 40°C. The supernatant was removed, frozen in liquid nitrogen, and stored at - 80°C. Aliquots of the supernatant (25 μl) were added to 1 ml GUS assay buffer (2 mM 4-methylumbelliferyl-beta-D glucuronide (Sigma, St. Louis, MO, USA) and 10 mM β-mercaptoethanol in extraction buffer), incubated at 37°C for 5, 35 and 95 minutes. Samples (200 μl) of this reaction were mixed with 800 μl stop solution (0.2 M Na_2_CO_3_), and the fluorescence measure with excitation at 365 nm and emission at 455 nm in a FLx 800 fluorescent microplate reader (BioTek Instruments, Winooski, VT). Protein content was measured at 595 nm using a commercial kit (Quick Start Bradford Protein Assay Kit; Bio-Rad, Hercules, CA, USA). Three replicates of 50 seedlings per replicate were measured at each time interval.

### Statistical analyses

The number of seedlings expressing GUS foci ≥ 2 mm was counted and analyzed using one-way or two-way ANOVA for a fixed effects model (Minitab. 15 software). If the counts were small (<10), tended toward a Poisson distribution and did not satisfy normality, the data were transformed using the square root transformation, and ANOVAs were performed on transformed data [31]. Mean comparisons were performed using The Tukey multiple comparisons at the 5% level (Minitab. 15). Replicates were performed in time, using different solutions, and the entire series of experiments were repeated with different seed lots and sometimes different operators, but gave similar results.

## Competing interests

The authors declare that they have no competing interests.

Most of the coauthors are members of the BioEnergy Science Center supported by the Office of Biological and Environmental Research in the DOE Office of Science.

## Authors' contributions

XC planned all of the experiments, carried out all of the experiments and drafted the manuscript. KB performed GUS staining, extracted mini-preps of genomic DNA and performed PCR screening of putative transformants. RE assisted XC with the experiments to optimize pretreatments and additives, and extracted maxi-preps of genomic DNA. HB assisted XC with the experiments to optimize pretreatments and additives, took photos and assisted with the tissue culture. JH and SA prepared fluorometric assays. JZ performed the statistical analysis and edited the manuscript. All authors suggested text modifications to earlier drafts of the manuscript, read and approved the final manuscript.
